# Are brief batteries able to identify aphasia in elderly people? A study with the ABLA Battery

**DOI:** 10.1590/2317-1782/e20250081en

**Published:** 2026-03-27

**Authors:** Karina Carlesso Pagliarin, Pâmela Lemes Rocha, Julia Prado Caieiro da Costa, Karin Zazo Ortiz

**Affiliations:** 1 Departamento de Fonoaudiologia, Universidade Federal de Santa Maria – UFSM - Santa Maria (RS), Brasil.; 2 Departamento de Fonoaudiologia, Universidade Federal de São Paulo – Unifesp - São Paulo (SP), Brasil.

**Keywords:** Aphasia, Aging, Language, Evaluation, Stroke

## Abstract

**Purpose:**

This study aimed to evaluate the performance of healthy elderly individuals and elderly individuals with post-stroke aphasia using the ABLA Battery - Brief Assessment of Language and Aphasias in order to verify if this brief battery is able to identify and characterize linguistic deficits in elderly individuals as well as to assess the effectiveness of this tool as a screening for aphasia.

**Methods:**

Sixteen neurologically healthy elderly individuals (Control Group- CG) and sixteen elderly persons with aphasia (Clinical Group EPWA) were assessed using the ABLA Battery. Data analysis was conducted using the Mann-Whitney U Test to compare performance on linguistic tasks between the two groups.

**Results:**

The Clinical Group scored significantly lower on all tasks of the ABLA Battery compared to the Control Group, with statistically significant differences (p<0.05).

**Conclusion:**

Aphasia negatively impacts the linguistic abilities of older adults, and the ABLA Battery was shown to be effective in promptly identifying these early changes.

## INTRODUCTION

According to the Pan American Health Organization^(1)^, by the year 2050, it is estimated that the proportion of the global population aged 60 years and over will nearly double, increasing from 12% to 22%. As of 2024, there is already a higher number of individuals aged 60 years or older compared to children under five years of age. This demographic shift underscores the process of population aging and highlights the importance of policies and programs designed to address the specific needs of this age group, encompassing areas such as health, well-being, and adequate care^(2)^.

The process of aging extends beyond the dimensions of chronological age, with psychological, biological, and social aspects also interacting and influencing the lives of older individuals^(3)^, with the Central Nervous System (CNS) being the most affected by aging^4^. Neuroimaging studies show that cognitive changes during aging correlate with neurobiological, neurophysiological, neurochemical and structural changes in the brain, such as reduced synapses, lower plasticity, and decreases in mass, volume and hemispheric asymmetry, among others^(4-6)^.

Several studies have demonstrated age-related changes in language processing among neurotypical older adults, with certain tasks being more susceptible to decline due to the aging process. A common language difficulty reported by older adults is the “tip-of-the-tongue” phenomenon, in which word retrieval becomes more challenging. This occurs because phonological and orthographic systems, which rely on rigid one-to-one connections, are more susceptible to failure. In contrast, semantic networks remain largely intact due to their rich interconnectivity, which enables compensation when certain links are weakened^(7)^.

In contrast, other language abilities—such as vocabulary, auditory comprehension, reading comprehension, and pragmatics—tend to remain relatively stable with age^(8-10)^. In this context, it is essential to understand how aging affects performance on language tasks in order to establish clinical parameters for assessing older adults with language impairments and to identify brief assessment batteries that may be useful for these purposes. Brief batteries are particularly important because, with advancing age, certain cognitive functions—such as processing speed, working memory, episodic memory, and inhibitory control—tend to decline^(5,10)^. Additionally, they are especially useful in hospital settings, as they enable the rapid assessment of language disorders in older adults.

Given the changes in the linguistic profile of elderly individuals that occur with advancing age, and considering other cognitive modifications observed in healthy aging, brief assessment batteries may be valuable for elderly patients with aphasia, as long as they can facilitate precise diagnosis and map linguistic changes within this population.

Aphasia is a language disorder characterized by a multimodal physiological inefficiency in verbal symbolic manipulations (e.g., association, storage, retrieval, and rule application). In its isolated form, it is caused by focal damage to the cortical and/or subcortical structures of the dominant hemisphere(s) for such symbolic manipulations^(11)^.

The ABLA Battery (Brief Assessment of Language and Aphasias) — previously referred in earlier studies as the Brief Version of the Montreal-Toulouse Language Assessment Battery^(12)^ - was developed from other screening tools^(13,14)^ and includes fundamental linguistic tasks for aphasia detection. It features stimuli controlled by linguistic parameters (phonology, syntax and semantics) but with a reduced number of stimuli per task.

Thus, it is necessary to determine how linguistic deficits related to aphasia in older adults can be accurately identified, taking into account the neurological changes already present in this population. These include the Hemispheric Asymmetry Reduction in Older Adults (HAROLD) effect^(15)^, the Compensation-Related Utilization of Neural Circuits Hypothesis (CRUNCH)^(16)^, among other previously mentioned changes.

This present study aimed to evaluate the performance of healthy elderly individuals and elderly individuals with post-stroke aphasia using the ABLA Battery in order to verify if this brief battery is able to identify and characterize linguistic deficits in elderly individuals as well as to assess the effectiveness of this tool as a screening for aphasia.

## METHODS

This study received approval from the Research Ethics Committee of Universidade Federal de Santa Maria under number 2.170.519, following the guidelines of the National Health Council resolution 466/12. Participants who voluntarily agreed to join the study signed the Informed Consent Form. This is an observational, analytical, case-control study with a quantitative approach.

### Participants

The sample comprised 16 elderly persons with aphasia (EPWAG) and 16 neurologically healthy elderly individuals (CG), all over the age of 60, with education levels ranging from illiteracy to higher education (M = 7.5; SD = 4.33). The groups were matched by age and education level. The assessment was comprehensively administered to all elderly participants, regardless of their educational background. Tasks requiring reading or writing were not administered to illiterate participants.

The neurologically healthy participants (CG) were recruited for convenience from university and social settings, while the patients in the EPWAG were recruited from a neurology clinic and a university hospital at two universities.

The inclusion criteria for both groups were: being over 60 years old and showing no signs of severe depression, as measured by the 15-Point Geriatric Depression Scale^(17,18)^, without signs of dementia, as measured by the Mini-Mental State Examination (MMSE), without a history of substance abuse, psychiatric disorders, and/or sensory disorders (uncorrected visual and/or auditory impairments), as assessed through the application of the Sociodemographic and Health Conditions Questionnaire^(19)^.

Participants in the EPWAG were required to be right-handed, have a diagnosis of aphasia based on the Montreal-Toulouse Language Assessment Battery - MTL-BR^(20)^, and a clinical history of stroke in the left hemisphere. Of these, two participants had chronic stroke, six had subacute stroke, and eight had acute stroke. The severity of aphasia ranged from moderate to severe.

### Procedures and instruments

All participants underwent the ABLA Battery, which evaluates, through 10 tasks, the linguistic components involved in oral expression and comprehension (words, simple and complex sentences), reading (words, pseudowords, and sentences), writing (words, pseudowords, and sentences), repetition, naming, and non-verbal praxis. The following is a brief description of each task:

1. Directed interview: Nine semi-structured questions are posed by the examiner (e.g., "What is your name?" "How are you feeling?" "What is the weather like today?") with the aim of qualitatively assessing oral expression and quantitatively evaluating auditory comprehension.
2. Auditory comprehension: Comprising a total of 11 items, this task assesses auditory comprehension through five words (using cards with six stimuli, including one target and five distractors) and six sentences (three simple and three complex, with a card containing four visual stimuli, only one of which is correct). Following the instructions, the participant must indicate the image corresponding to the examiner's statement.
3. Written comprehension: This task consists of 11 items assessing graphic comprehension of five words (cards with the word written in block letters and six images, with only one correct target) and six sentences (three simple and three complex, with cards containing the target sentence and four images, only one of which is correct). After silently reading the written word or sentence, the participant must point to the corresponding image.
4. Copy: The participant is asked to copy a sentence using their own handwriting, in order to assess their ability to recognize and reproduce letters.
5. Dictation: The examiner dictates two words, one pseudoword and one sentence, for the participant to write, evaluating their ability to produce written representation in response to auditory stimuli.
6. Repetition: The participant is required to repeat a total of 12 items, including words, a pseudoword, and a sentence, in order to assess their oral repetition ability.
7. Reading aloud: To assess the ability to read via both lexical and sublexical routes, the participant must read a total of 12 stimuli, including regular and irregular words, pseudowords, and a sentence.
8. Oral naming: The participant is required to name 12 black-and-white pictures, including nine nouns and three verbs, to evaluate lexical access via visual input.
9. Automatic speech: Comprising automatic sequences (counting aloud from one to twenty and singing the song "Happy Birthday"), this task evaluates the ability to evoke linguistic automatisms.
10. Non-Verbal Praxis: The participant is required to perform six non-verbal praxic gestures as instructed verbally, in order to assess the ability to produce isolated and sequential movements

For result interpretation, 1 point is assigned for each correct response in the auditory comprehension and written comprehension tasks. In the directed interview and oral naming tasks, scores can be 0 (incorrect), 1 (incomplete or close to the target), or 2 (correct). The automatic speech task is analyzed for both form (occurrence of phonemic errors) and content (occurrence of omissions). In the copying task, 1 point is assigned for each correctly written word in the sentence, totaling up to 4 points. In the dictation, repetition, and reading aloud tasks, 1 point is given for each correctly written word and pseudoword, and 1 point for each word in the sentence, totaling up to 4 points. In the non-verbal praxis task, scoring is as follows: 0 points are given for incorrect movements or no response. If the participant provides a partial response, 1 point is awarded. If the movement is performed correctly after a demonstration, 2 points are assigned. For movements correctly executed after practice, 3 points are given. Finally, 4 points are awarded for movements performed correctly without practice, based solely on verbal instruction.

### Data analysis

The data were analyzed using the Mann-Whitney U Test to compare the CG and the EPWAG, with significance considered for p-values less than 5%. The analysis was conducted with the assistance of SPSS version 30.0 for MAC.

## RESULTS

In the CG, the mean age was 69.06 years (SD=7.99 years), while the mean education level was 6.00 years (SD=3.44 years). The mean age in the EPWAG was 69.94 years (SD=4.63 years), and the mean education level was 5.75 years (SD=3.66 years). The groups were matched by age (p =0.707) and education (p = 0.844).

Among the EPWAG, two had anomic aphasia, one had Broca’s aphasia, four had global aphasia, five had mixed aphasia, and four had transcortical motor aphasia.

Table 1 presents the mean performance scores on the different tasks of the ABLA Battery, comparing the results obtained by participants from the control group with those from the clinical group.

**Table 1:** Comparison of ABLA Battery performance between the control group and the clinical group

**Task/ maximum score**	**Group**	**N**	**Mean**	**Standard deviation**	**p-value**
Directed Interview/18	Clinic	16	13.19	5.89	0.002
	Control	16	17.75	1.00	
Oral Comprehension/11	Clinic	16	6.00	3.81	≤ 0.001
	Control	16	9.38	1.50	
Written Comprehension/11	Clinic	16	4.50	4.24	≤ 0.001
	Control	16	9.25	2.20	
Copy/4	Clinic	16	1.56	1.75	≤ 0,001
	Control	16	3.69	1.01	
Dictation/7	Clinic	16	1.75	2.67	≤ 0.001
	Control	16	4.88	2.06	
Repetition/12	Clinic	16	7.25	5.33	0.004
	Control	16	11.63	1.02	
Reading Aloud/12	Clinic	16	5.44	5.08	≤ 0.001
	Control	16	11.69	0.60	
Oral Naming/24	Clinic	16	11.81	10.25	≤ 0.001
	Control	16	22.38	2.21	
Automatic speech (form)/4	Clinic	16	2.13	1.58	≤ 0.001
	Control	16	3.94	0.25	
Automatic speech (content)/4	Clinic	16	2.44	1.67	0.002
	Control	16	4.00	0.00	
Non-Verbal Praxis/24	Clinic	16	17.25	7.27	0.002
	Control	16	23.38	1.25	

It can be observed at Table 1, that the groups differed in all tasks of the ABLA Battery, with the clinical group showing worse performance.

## DISCUSSION

The results of this study demonstrate significant differences in linguistic performance between healthy elderly individuals and those with post-stroke aphasia across all linguistic tasks assessed by the ABLA battery. These findings suggest the feasibility of using brief instruments for evaluating aphasia in the elderly. Such findings will be discussed further.

Initially, it is noteworthy that, despite the elderly individuals being assessed using a brief battery, several linguistic tasks important for the diagnosis of aphasia were included^(13,21,22)^. Even with all the linguistic tasks included, what truly made the ABLA a brief instrument was the number of stimuli per linguistic task. Thus, we can hypothesize that the limited number of stimuli per task does not hinder accurate diagnosis and, on the contrary, may be beneficial for diagnosing elderly individuals, taking into account the cognitive changes associated with healthy aging, such as those related to processing speed, working memory, episodic memory, and inhibitory function^(23,24)^.

Although there were statistically significant differences in all linguistic tasks, several considerations regarding the obtained results are warranted.

In the directed interview, questions of varying complexity were asked, and scores were assigned based on participants’ comprehension. In this task, we observed the smallest difference between the two groups, which may suggest that the use of more everyday, familiar questions (as opposed to those found in specific comprehension subtests) facilitated understanding in older adults. Although this task is considered complex—given the range of possible expressive impairments in persons with aphasia (PWA)—participants were still required to demonstrate their comprehension at least partially through oral responses. Interestingly, the EPWAG in this study performed slightly better on this task. Indeed, although aging is associated with changes in language processing^(25)^, including alterations in oral comprehension^(26)^, previous studies have shown that such changes do not appear to significantly impact everyday communication activities^(27,28)^, which tend to remain relatively stable. For this reason, the directed interview may have been the easiest task, even for EPWAG.

Important tasks for evaluating oral communication, such as oral comprehension and repetition, while also capable of differentiating healthy elderly individuals from EPWAG, appeared to be less compromised than tasks requiring some degree of reading and writing participation, such as copying, writing from dictation, and reading aloud. This is likely due to an overlap between low educational attainment and the occurrence of brain injury, which may have exacerbated the reading and writing difficulties already experienced by some participants, considering that the average formal education of the participants in this study was only six years.

Writing is a complex process that involves linguistic, spatial, and perceptual (visual) systems. It relies on knowledge of the graphemic system, the ability to map phonemes onto graphemes, and/or direct access to the orthographic lexicon. As a result, a variety of impairments may occur, affecting sublexical and/or lexical routes and often accompanied by spatial or perceptual difficulties.

Additionally, reading deficits may impact both the lexical and perilexical routes. The lexical route appears to be more vulnerable to failure, particularly in individuals with lower educational levels. In contrast, the perilexical route may struggle to accurately process pseudowords, resulting in lexicalization due to their resemblance to real words. Beyond these potential failures within the reading system, reading aloud also depends on the motor system to plan the articulation of phonemes — a function frequently impaired in individuals with aphasia^(29)^.

It is important to highlight that, although the ABLA is a simple battery with few stimuli per task, it includes stimuli that allow for the tracking of reading and writing pathways, such as pseudowords and irregular words. Nonetheless, the difference found between the two groups identified for these tasks in this study confirms the findings of a previous study that observed that the formal evaluation of aphasia (test) is able to distinguish the neurological lesion effect from the effect of low education in post-stroke PWA^(30)^. However, the previous study was conducted using a comprehensive language battery applied to young adults with low education post-stroke, unlike this study, which utilized a brief battery in elderly individuals.

The ABLA subtests for oral and written comprehension, as well as for repetition and reading aloud, are matched in terms of the number of stimuli and their complexity. For this reason, the results obtained in this study reinforce that difficulties were greater in reading and writing tasks for these patients.

Regarding the oral naming task, it effectively differentiated the two groups. Although PWA, regardless of the type and severity of aphasia, experienced lexical access difficulties, it was necessary to investigate whether this subtest could differentiate healthy elderly individuals from PWA. This is because the task involves response time, and we know that lexical access activities are also slower for healthy elderly individuals. During the aging process, word finding difficulty is one of the most common age-related complaints. The individual normally says that they know what they want to say but are unable to find the appropriate word. In fact, the activation of phonological representations of the word is a very complex process that demands connections established are “one to one”, and, in this sense, more susceptible to failure. A transmission deficit in single connection hampers recall of the phonology representations. On the other hand, semantic networks are normally more preserved in healthy aging, allowing a deficit in one connection to be compensated by other connections sharing the same semantic field^(26)^ Although some difficulties in lexical access in neurotypical elderly, this task was able to discriminate between both groups.

In turn, the differences found in the non-verbal praxis task suggest that some of the EPWAG may present a manifestation of apraxia associated with aphasia. In fact, the association between the two syndromes can be expected in approximately 30% of cases^(31)^.

Considering the findings of this study, all tasks of the ABLA Battery were able to differentiate healthy elderly individuals from EPWAG. Additionally, it was observed that older adults in the control group obtained the highest scores on the ABLA Battery tasks (Table 1). In the automatic speech task, a ceiling effect was observed and it is well known that the verbal information required in the automated language task is one of the most resistant to aging^(32)^. These results suggest that the language tasks assessed by the ABLA Battery are not affected by the aging process.

The development of brief language batteries is particularly complex because, while they need to be concise, they must also assess all important aspects for diagnosis. Regarding language, given the complexity of this cognitive function, creating brief batteries that meet this demand is a challenge. Even with similar ages and educational levels, the EPWA group demonstrated lower performance in all tasks, indicating the impact of aphasia on linguistic abilities. In this context, this study demonstrated that the ABLA can be a useful instrument for assessing aphasia in the elderly.

One of the main limitations of this study was the small sample size and the heterogeneity of the EPWA group. Future studies with larger and more homogeneous subgroups of patients with different types of aphasia are needed to better elucidate the role of the battery in this diagnosis. Moreover, it remains necessary to investigate the construct validity between the ABLA and comprehensive language batteries. Future research could also explore whether the ABLA is useful for assessing language disorders in other neurological syndromes in adults and older adults.

## CONCLUSION

The results of this study demonstrate that the ABLA Battery effectively differentiates healthy older adults from EPWA, accurately identifying and characterizing linguistic deficits. Participants in the control group performed within normal limits on all tasks, indicating that the assessed language abilities are not significantly affected by aging. Furthermore, due to its brevity, the ABLA Battery shows promise as a practical tool for initial language screening in clinical and hospital settings.
